# The Architecture of Thiol Antioxidant Systems among Invertebrate Parasites

**DOI:** 10.3390/molecules22020259

**Published:** 2017-02-10

**Authors:** Alberto Guevara-Flores, José de Jesús Martínez-González, Juan Luis Rendón, Irene Patricia del Arenal

**Affiliations:** Departamento de Bioquímica, Facultad de Medicina, Universidad Nacional Autónoma de México (UNAM), Apartado Postal 70-159, 04510 Mexico City, Mexico; guevarafa@yahoo.com.mx (A.G.-F.); prometeo_quetzalcoatl@ciencias.unam.mx (J.J.M.-G.); jrendon@bq.unam.mx (J.L.R.)

**Keywords:** antioxidant systems, parasites, thiol-dependent peroxidase, disulfide reductase, redoxin, thiol, redox mechanisms

## Abstract

The use of oxygen as the final electron acceptor in aerobic organisms results in an improvement in the energy metabolism. However, as a byproduct of the aerobic metabolism, reactive oxygen species are produced, leaving to the potential risk of an oxidative stress. To contend with such harmful compounds, living organisms have evolved antioxidant strategies. In this sense, the thiol-dependent antioxidant defense systems play a central role. In all cases, cysteine constitutes the major building block on which such systems are constructed, being present in redox substrates such as glutathione, thioredoxin, and trypanothione, as well as at the catalytic site of a variety of reductases and peroxidases. In some cases, the related selenocysteine was incorporated at selected proteins. In invertebrate parasites, antioxidant systems have evolved in a diversity of both substrates and enzymes, representing a potential area in the design of anti-parasite strategies. The present review focus on the organization of the thiol-based antioxidant systems in invertebrate parasites. Differences between these taxa and its final mammal host is stressed. An understanding of the antioxidant defense mechanisms in this kind of parasites, as well as their interactions with the specific host is crucial in the design of drugs targeting these organisms.

## 1. Introduction

During the Precambrian era, about 2.5 billion years ago, the appearance of oxygenic photosynthesis gave place to a significant increase in the oxygen content in the oceans, producing the oxidation of dissolved iron and leading to its precipitation as ferric oxide [1,2]. After exhaustion of the reduced iron and compounds related to it, the oxygen concentration in the atmosphere began to increase, reaching 21% abundance at present and producing a diversity of environments subjected to different oxidizing conditions [3].

**Figure 1:**
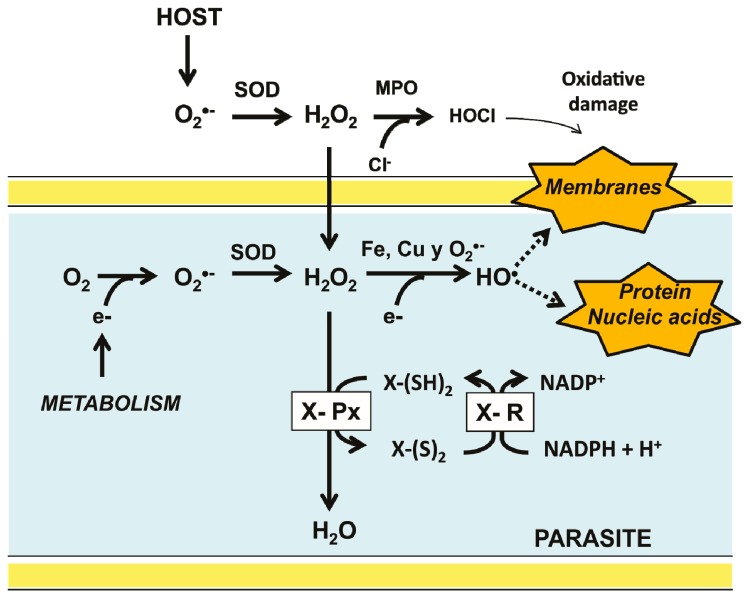
Major pathways for ROS production in invertebrate parasites inside its host and the antioxidant defense system. Superoxide anion (O_2_^−^) is produced as a metabolic byproduct in both the parasite and its host. To contend with the deleterious effect of its ROS derivatives, a typical antioxidant defense system is present in the parasite. It is constituted by a thiol-dependent peroxidase (X-Px) which transfers electrons from a reduced donor substrate (X-(SH)_2_) to H_2_O_2_. The resulting oxidized form of the substrate [X-(S)_2_] is then reduced by a specific disulfide reductase.

Some of the earlier organisms were capable to survive and adapt to the presence of oxygen, leading to the appearance of the aerobic metabolism [4]. As a result, new metabolic pathways arose, producing a variety of new compounds. Eventually, the emergence of cell compartmentalization gave place to the first eukaryotic organisms [1,5]. However, as a result of the use of molecular oxygen as the final acceptor of the electrons derived from food, a variety of collateral compounds, known as reactive oxygen species (ROS) are produced. These include the superoxide anion (O_2_^−^) which is enzymatically dismuted into hydrogen peroxide (H_2_O_2_) by superoxide dismutase (SOD) [6]. Although not a radical, H_2_O_2_ is potentially able to generate the radical hydroxyl (^•^OH) through either the Haber-Weiss or the Fe^2+^-dependent Fenton reactions (Figure 1). At low concentrations, some ROS are involved in signaling pathways, participating in the regulation of essential cell process, such as metabolism and transcription [7,8,9]. However, at high concentrations, ROS are potentially harmful for living organisms, acting as unspecific oxidants of proteins, lipids, and nucleic acids. As a result, oxidative stress is produced [10].

To contend with such oxidant stress, aerobic organisms have evolved both enzymatic and non-enzymatic antioxidant systems. In the former SOD, catalase (CAT) as well as the glutathione and thioredoxin dependent enzyme systems [11] are included. The two latter are critically dependent on the redox properties of the thiol group for its function. Such systems are essential in the maintenance of the redox homeostasis in cells. Their alteration can lead to an increase of oxidized proteins, resulting in a decrease or the total loss of its function. In many cases, such oxidation make them prone to degradation [12]. In this sense, thiol groups are directly involved in the process of protein degradation through their participation in the following events: (i) ubiquitination of proteins depends on the formation of thioester bonds between ubiquitin and a thiol group of E1 and E2, so that the oxidation of thiol groups would result in alterations of the process [13,14]; (ii) in the 20S core particle of the proteasome two disulfide bonds are present, and its activity could be modulated by a redox mechanism like glutathionylation [15]; the presence of an oxidized cysteine in a *N*-degron of some proteins is recognized by a specific ubiquitin ligase E3 [16].

In the non-enzymatic antioxidant systems ascorbate, retinol derivatives and tocopherol are included, as well as low molecular weight thiols, particularly glutathione [17]. Although in most of the living phyla GSH is the main antioxidant compound, in some representatives of prokaryotes and fungi alternative thiol-containing compounds have been found [18]. Outstanding are mycothiol in Actinobacteria [19] and bacilithiol in species of *Bacillus*, *Streptococcus*, and *Staphylococcus* [20].

Parasitic organisms, unlike its free-living counterparts, are under continuous attack by the chemical defense mechanisms of its host, which produce compounds such as H_2_O_2_ and hypochlorous acid (HOCl) to contend with invaders [21,22] (Figure 1). Furthermore, the complexity of the life cycle of a diversity of parasites expose them to variations in oxygen levels and hence to a broad range of oxidative stresses. Thus, the development of parasitic protozoa inside its host involves different stages, occurring in both intra- and extracellular compartments. Hence, they are exposed to a broad range of oxygen tensions [23,24]. Similarly, some parasitic invertebrates (e.g., representatives of the phyla Platyhelminthes and Nematoda) require access to different compartments inside the host. Hence, the antioxidant defense systems of parasites are critical for their survival (Figure 1). In mammals, the harmful effects of H_2_O_2_ are preventable by CAT, however, there are no evidence for the presence of such an enzyme in parasites [21,25]. In the latter the disposal of H_2_O_2_ is based on peroxidases which are dependent on either GSH or Trx [26]. By contrast, in some parasitic protista (Kinetoplastida), the antioxidant defense system is based on tryparedoxin peroxidase, which is dependent on trypanothione, a low molecular weight thiol [27]. As a result of the activity of such enzymes, the oxidized states of glutathione (GSSG), thioredoxin (Trx-S_2_), and trypanothione (T-S_2_) are produced. The reduced state is regenerated by specific NADPH-dependent disulfide reductases [28].

Because parasitism is an extended ecological interaction between organisms, in the present review we focus on invertebrate parasites for which mammals are an essential host in its life cycle. Therefore, vertebrate parasites such as *Lampetra japonica* (a parasite lamprey) [29,30], *Rhodeus* (bitterlings), or *Vandellia cirrhosa* (a parasite catfish) [31] have been excluded. Thus, the major antioxidant defense systems of animal endoparasites belonging to the protista and invertebrate metazoan are the focus of the present work. Information about those genera involved in human parasitic diseases (e.g., *Entamoeba*, *Plasmodium*, *Taenia* and *Schistosoma*) is particularly stressed.

## 2. Major Redox Substrates

### 2.1. Generalities

As a result of various metabolic processes, mainly the mitochondrial respiratory chain, the radical anion superoxide (O_2_^−^) is produced [32]. A first defense mechanism to contend with involves its elimination through dismutation into H_2_O_2_. The enzyme responsible of such reaction is SOD, which is present in both prokaryotes and eukaryotes [33]. In the course of the reaction, electrons are provided by a substrate whose redox activity depends on the presence of a sulfhydryl group (-SH) [34]. Although the chemical nature of such reducing compounds is diverse, they can be grouped into two families:

(i) Low molecular weight thiol compounds, such as cysteine (Cys), glutathione (GSH), ovothiol (OSH), and trypanothione (TSH) [17].
(ii) Thiol-containing proteins, which includes thioredoxin (Trx), tryparedoxin (TXN), plasmoredoxin (Plrx), as well as the dithiol and the monothiol variants of glutaredoxin (Grx) [35,36].

In addition to its participation in the antioxidant defensive mechanisms, some of these reducing compounds are involved in the reduction of ribonucleotides [37,38], as well as in redox signaling process [7,8,9].

### 2.2. Characteristics of Thiol-Containing Redox Substrates

#### 2.2.1. Low Molecular Weight Thiols

##### Cysteine (Cys)

A major physiological event which is critical for the survival of all living organisms is protein folding. This process involves Cys residues, which become oxidized through the formation of disulfide bonds. In eukaryotes protein folding occurs in the endoplasmic reticulum [39], while in prokaryotes the periplasmatic compartment is involved. The correct formation of the protein disulfide bonds, either reversible or irreversible, depends on protein disulfide isomerases (PDI) [40]. In some cases, the formation of mixed disulfides is necessary, such as those involving GSH [41].

Due to its chemical properties, Cys is involved in diverse physiological processes, such as protein folding [42], the catalytic action of enzymes which are dependent on the presence of a -SH, as well as in the maintenance of redox homeostasis [43]. In this latter role, Cys plays a major role as a functional component of reducing substrates (e.g., GSH, Trx, and TSH) [44].

The biosynthesis of Cys occurs through either the de novo or the trans-sulphuration pathways [44]. In some parasitic Protista, such as *Trypanosoma cruzi*, both biosynthetic pathways are present [45], while in others (*Trichomonas vaginalis*), only one biosynthetic pathway is functional [46]. Interestingly, in *Plasmodium* the capacity for the formation of Cys is lacking [47]. In some species with the ability for Cys biosynthesis a requirement for additional Cys have been noted [48].

##### Selenocysteine (Sec)

In addition to Cys, selenocysteine (Sec in the three-letter code or U in the one-letter code) plays a crucial role in enzyme-mediated redox reactions [49] with significant differences as compared with cysteine:

(i) Structurally, cysteine is characterized by the presence of a sulfur atom, which is critical for its biological functions. By contrast, in Sec a selenium atom replaces Cys [49].
(ii) Unlike Cys, Sec does not exist as a free amino acid within the cell. Instead, it is synthesized on a specific tRNA [50,51].
(iii) Selenocysteine, like canonical amino acids, is incorporated into proteins during the translation process. However, its insertion requires a specific UGA codon (normally a termination codon) located inside the open reading frame of the corresponding gene [52]. To be recognized as Sec instead of a stop signal of translation, a specific context is required which is given by trans-acting translation factors [53] that can recognize and interact with a cis-acting stem-loop structure in a selenoprotein mRNA. This structure has been named selenocysteine insertion sequence (SECIS) and is located immediately after the UGA codon within the coding region in eubacterias. By contrast, in archaeas and eukaryotes the SECIS element is located at the 3′ untranslated region of mRNA [54]. The SECIS element is an essential factor for incorporation and recruitment of the Sec-tRNA [55,56].
(iv) In those proteins in which Sec has been incorporated, a unique of such residue is present per subunit. By contrast, the number of Cys residues found in proteins is variable, and can represent a significant fraction of the total amino acid residues (e.g., albumin). To date, the only exception is represented by the vertebrate selenoprotein P, in which 10 to 17 Sec residues are present [57].
(v) As regard the reactivity of selenocysteine, this amino acid may be susceptible to redox phenomena similar to those of cysteine. However, due to the electronic configuration of selenium, the conjugate base of selenocysteine (selenolate anion Se^−^) is more stable than the corresponding conjugate base of cysteine (thiolate anion S^−^) and hence selenol (-SeH) is more acidic than thiol (-SH) (Sec p*K*_a_ = 5.2 vs. Cys p*K*_a_ = 8.3). Therefore, at physiological pH the selenol group of selenocysteine is present in its selenolate form [49], which makes it more reactive during catalysis than its protonated thiol counterpart, thereby increasing the catalytic efficiency of selenoenzymes [58].

##### Glutathione (GSH)

Glutathione (γ-l-glutamyl-l-cysteinylglycine) is a tripeptide with a molecular mass of 307 Da constituted by glutamate, cysteine, and glycine [59]. The presence of a gamma-glutamyl peptide bond involving the glutamate and cysteine residues makes it resistant to hydrolysis by peptidases [60]. GSH is involved in a diversity of cell processes, which can be summarized as follows:

(i) Redox homeostasis of all cell compartments, including the removal of ROS and the regeneration of the reduced state of ascorbic acid [61,62].
(ii) Fill intermediaries of GSH and transport of amino acids through the γ-glutamyl cycle [63].
(iii) Formation of deoxyribonucleotides. In this process, GSH acts as a reducing compound by transferring electrons to Grx and then ribonucleotide reductase (RR) [64].
(iv) Removal of xenobiotic compounds. In this function, GSH works either by increasing the solubility of potentially harmful foreign substances [65], or through its covalent conjugation to xenobiotic compounds by glutathione S-transferases (GST) [66].
(v) Recovery of the native conformation of proteins damaged during an oxidative stress. This process requires the participation of Grx [67].
(vi) Cell signaling. The participation of GSH as mediator in cell signaling processes involves its reversible covalent binding to a diversity of proteins through glutathionylation [68].

**Figure 2:**
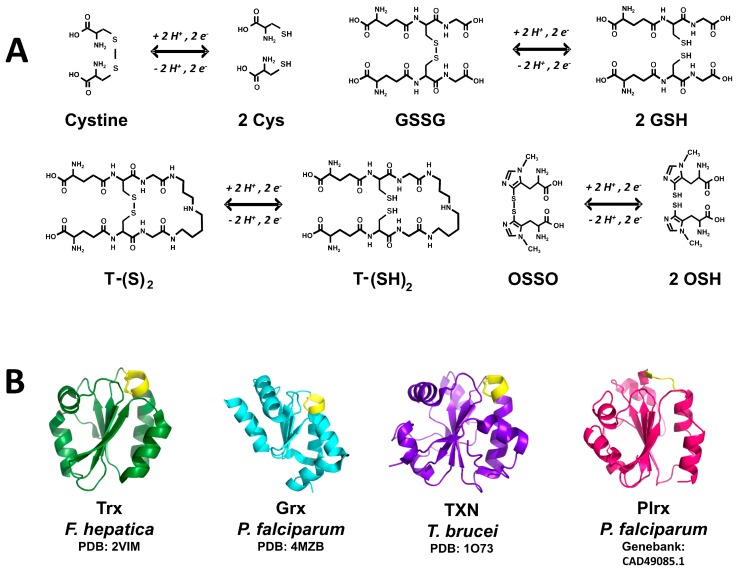
Diversity of thiol-containing redox substrates in invertebrate parasites. (**A**) The thiol-containing substrates of low molecular weight cysteine (Cys), glutathione (GSH), trypanothione (T-SH_2_) and ovothiol (OSH) are shown. Both the reduced and the disulfide form of each compound is shown; (**B**) Schematic representation of the three-dimensional structure of the thiol-containing substrates of protein nature as thioredoxin (Trx), glutaredoxin (Grx), tryparedoxin (TXN), and Plasmoredoxin (Plrx). Active sites are shown in yellow. For Trx, Grx, and TXN the corresponding PDB file is indicated. The model corresponding to Plrx was constructed from the amino acid sequence data base by using the automatized SWISS-MODEL server (http://swissmodel.expasy.org). Models were drawn with PyMOL.

In most of the cell functions in which GSH is involved, its thiol group is oxidized into a disulfide bond with other glutathione molecule, producing oxidized glutathione (GSSG) (Figure 2A). In the cytosolic compartment of mammalian cells the GSH/GSSG concentration ratio is about 100 under normal physiological conditions [65]. Such ratio is the main determinant of the cellular redox potential. However, in cell compartments other than the cytosolic (e.g., endoplasmic reticulum, vacuoles and mitochondria) the existence of independent pools of GSH have been reported [62], in which the above noted ratio can be significantly different [69]. Such differences in the GSH/GSSG concentration ratio reveals variations in the intracellular redox environment [70].

Although in most organisms GSH represents the more abundant low molecular weight thiol, whose concentration is in the range 0.2 mM to 10 mM [71], there are alternative thiol compounds. Thus, in *Mycobacterium tuberculosis* the presence of micothiol (MSH) has been reported [19], while in representatives of the genera *Bacillus* and *Staphylococcus* bacilithiol is present [20].

##### Trypanothione (TSH)

Trypanothione represents a dithiol variant of glutathione (Figure 2), in which two GSH molecules are covalently joined through amide bonds to a spermidine molecule. The compound was discovered in 1985 by Fairlamb [72]. The enzyme trypanothione synthase is involved in its biosynthesis. The monothiol precursor glutathionil-spermidine is a better nucleophilic agent as compared with TSH, being incorporated easily into mixed disulfides [73]. In this sense, the p*K*_a_ value of 7.4 for the nucleophilic thiol of TSH makes it more reactive under physiological conditions as compared with GSH [17]. TSH represents the main electron donor to tryparedoxin, which is involved in the removal of hydroperoxides, a process dependent on peroxiredoxins [74,75].

##### Ovothiol (OSH)

The existence of this compound was reported first in sea urchin eggs [76]. It is a mercaptohistidine with a variable number of methyl groups at the amino group of the amino acid. Depending on the absence or the presence of methyl groups, different OSH variants can be found. Thus, OSH-A lacks methyl groups, while OSH-B and -C are characterized by the presence of one or two methyl groups, respectively. A striking feature of OSH is the unusually low p*K*_a_ value of 1.42 for it’s -SH [77], which thus exists in the thiolate ionized state (-S^−^) at pH values above 2. OSH is involved in H_2_O_2_ reduction, leading to the disulfide form of the compound (OSSO) (Figure 2) [78]. The latter can be reduced by either TSH (see below) or GSH [79]. To date, there are no reports on the existence of an ovothiol reductase.

#### 2.2.2. Redox Protein Substrates (Redoxins)

##### Thioredoxin Superfamily

The most representative member of this superfamily of proteins is thioredoxin (Trx), which was discovered in *E. coli* as a cofactor needed for ribonucleotide biosynthesis [80]. At present, additional Trx-like proteins have been added to the superfamily, including glutaredoxin (Grx), Tryparedoxin (TXN), and Plasmoredoxin (Plrx) (Figure 2B), as well as protein disulfide isomerase (PDI) [81]. They are small monomeric proteins featured by the presence of the “thioredoxin fold”. The main characteristics of the superfamily are:

(i) A protein core constituted by a ß-sheet sandwiched between a variable number of α helix segments. In some cases, such as PDI, an additional Trx-like segment can be present [40].
(ii) A common CXXC redox active motif located at the C-terminal end of a β-sheet segment and the start of the α-helix 1. In some representatives of the family (e.g., an isoform of Grx), the C-terminal cysteine residue of the redox motif can be absent.
(iii) The presence of a conserved *cis* proline (*cis*-Pro) located in a fork at the N-terminal end of a β-sheet segment (β2 for Trx, β6 de TXN) [82]. The *cis*-Pro containing fork is located near to the CXXC redox motif, and plays an essential role both in the structural stability and in the ability for binding proteins [83].

The catalytic mechanism of most of the members of the Trx superfamily involves both cysteine residues of the CXXC redox motif. In the catalytic cycle the N-terminal cysteine residue, called the catalytic Cys, acts as a nucleophilic reagent. Its p*K*_a_ value of 7.2 suggests at physiological pH values a significant fraction of the sulfhydryl group of such Cys is in the thiolate dissociated state –S^−^ [84]. The anionic form stabilizes through hydrogen bonds with basic residues [85]. As for the C-terminal cysteine residue, its presence is essential in those reactions involving disulfide reduction (Figure 2B). The nature of the residues located between both catalytic Cys depends on the specific protein.

**Figure 3:**
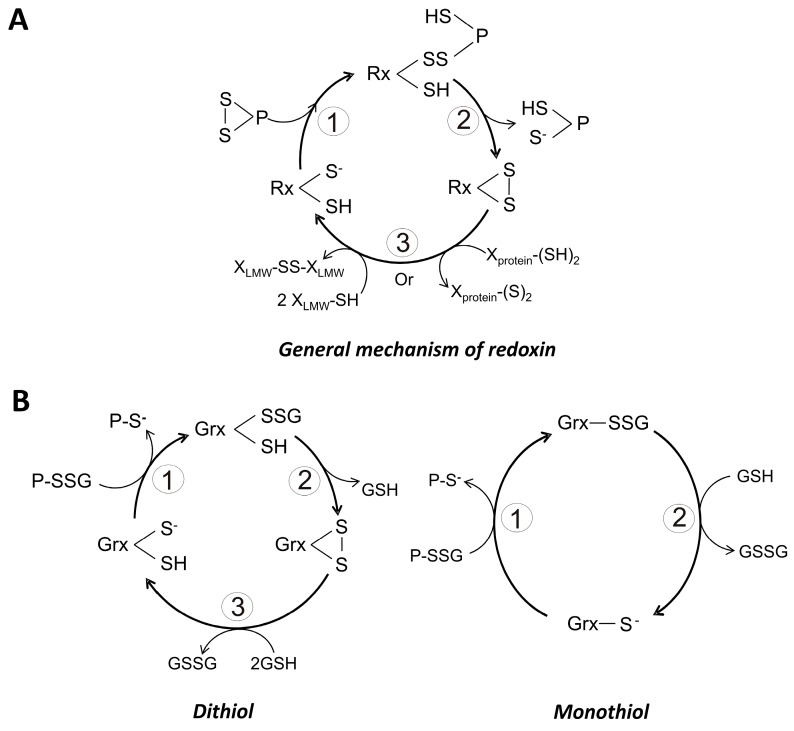
General catalytic mechanisms for monothiol and dithiol redoxins. (**A**) General catalytic cycle for the reduction of a protein disulfide by a redoxin. The potential electron donor for the redoxin can be a low molecular weight thiol (LMW-SH) or a high molecular weight thiol (X-protein (SH)_2_; (**B**) General catalytic cycle for the deglutathionylation of a protein-glutathione mixed disulfide by either a dithiol or a monothiol glutaredoxin. In this case, GSH is the obligate electron donor.

The chemical reactivity of the catalytic Cys can be modified by the residue located N-terminal to the above mentioned *cis*-Pro [86]. Such residue can be of hydrophobic nature, mainly isoleucine or valine, and acts through hydrophobic interactions with those residues located between the catalytic Cys. In some members of the superfamily, the N-terminal residue is a threonine, which contributes to the stabilization of the catalytic thiolate through the formation of a hydrogen bond [85]. When the *cis*-Pro residue is changed, the formation of mixed disulfides with protein substrates is favored [87]. Interestingly, for human Trx such amino acid replacement leads to an inability of the protein for binding metal ions [86]. The general catalytic mechanism of redoxins in the reduction of protein disulfides is as follows (Figure 3A):

(i) The thiolate form of the catalytic cysteine (S_C_H) performs a nucleophilic attack on a sulfur atom of a disulfide bond in the protein substrate, generating an intermolecular redoxin-protein mixed disulfide.
(ii) Through a second nucleophilic attack involving the resolving cysteine (S_R_H) on the mixed disulfide the reduced state of the substrate is produced. As result of this process, an intramolecular disulfide bond in the redoxin is produced.
(iii) The resulting disulfide bond in the redoxin is reduced either by a NADPH-dependent specific reductase or through the participation of reduced glutathione. This last step regenerates the biologically useful dithiol form of the redoxin.

A diversity of oxidized proteins can serve as targets of redoxins. In the specific case of reduced Trx, its reducing equivalents can be transferred to peroxidases, while TSH can reduce chemically oxidized molecules such as OSSO.

##### Glutaredoxin (Grx)

Glutaredoxins are a set of low molecular weight redox-active proteins involved in both disulfide reduction and thiol-disulfide exchange reactions. They have a wide distribution in the living world, both in prokaryotes and eukaryotes. In the latter, Grxs are present in the cytosolic, nuclear, and mitochondrial compartments [88]. Grxs are components of the glutathione redox system, which also includes GSH, glutathione reductase (GR) and glutathione peroxidase (GPx) [70]. Grxs are critically dependent on GSH for their function. They have the ability for dimerization. In this sense, it has been reported that by interacting with iron-sulfur redox centers (2Fe-2S), Grx can aggregate into dimers and tetramers, leading to its inactivation [89]. In this process GSH plays a critical role [90].

Grxs are involved in redox signaling pathways either by reducing protein disulfides in a GSH-dependent mode or through deglutathionylation of covalently modified proteins. The first process makes possible the reactivation of oxidized enzymes, while the glutathionylation is particularly important under oxidative stress conditions, where a significant number of proteins becomes glutathionylated as a mechanism for protect them from over-oxidation [41].

Based on the number of cysteine residues present in the redox active motif, Grx are grouped as either monothiol (1-Cys-Grx) or dithiol (2-Cys-Grx) dependent Grx. In the first group, only the N-terminal Cys of the redox motif is present, and the sequence CGFS is generally found. By contrast, in the dithiol Grx both the N- and C-terminal Cys residues are present, and a sequence CPYC is typical [91]. In both cases GSH is required for its reduction [92]. There are also mechanistic differences between both types of Grx. Thus, the monothiol variant is capable to catalyze only thiol-disulfide exchanges (Figure 3B), being dependent on GSH for the completion of the catalytic cycle. By contrast, dithiol Grx participate in the reduction of disulfides (Figure 3B), and no additional reducing compound is needed in the process. In this case, GSH acts to regenerate the reduced form of the protein. As predicted, the p*K*_a_ value of the catalytic Cys is low, making it a good nucleophilic reagent. Values as low as 3.5 have been reported for the p*K*_a_ of the N-terminal Cys of the redox motif [88]. The mechanistic details of both 1-Cys-Grx and 2-Cys-Grx are summarized at Figure 3B.

##### Thioredoxin (Trx)

The archetypical redoxin (Figure 3A) is capable to participate in dithiol-disulfide redox reactions, as exemplified by Trx. This latter is a low molecular weight protein (~12 kDa) with a variant of the CXXC redox motif in its catalytic center. Trx is present in cytosol, mitochondria, nucleus and chloroplasts. It has been found even in the extracellular environment [36]. Trx is a multifunctional protein, being involved in the following cellular process:

(i) Synthesis of deoxiribonucleotides [38].
(ii) Detoxification of H_2_O_2_ through the activity of peroxiredoxins [93].
(iii) Regulation of the activity of transcription factors such as AP-2 and NF-κB [94].
(iv) Regeneration of methionine sulfoxide acting as an electron donor to methionine sulfoxide reductase (MSR) [95].
(v) Under oxidative stress conditions Trx is secreted, then acting as a cytosine [96].
(vi) Its active CXXC redox motif can serve as a redox rheostat [97].

In addition to the redox active Cys, in Trx additional Cys residues can be present. These latter can be post-translationally modified and are important in the regulation of Trx activity. In this sense, in human Trx an intramolecular disulfide bond involving both Cys62 and Cys69 can be formed, resulting in inactivation of the protein [98]. It has been proposed such inactivation leads to an inability of recognition by its specific reductase [99]. On the other hand, the formation of intermolecular disulfides in human Trx leads to dimeric aggregates. In this case, Cys73 of both monomeric units could be involved. Under reducing conditions such covalent cross-linking of Trx is not feasible, although its aggregation into dimers through non-covalent interactions is possible. Additional potential modifications of the Cys involves either nitrosylation or glutathionylation [100], resulting in changes in Trx activity. These latter covalent modifications have been reported in Trx from a diversity of sources [101].

##### Tryparedoxin (TXN)

Tryparedoxin represents a variant of Trx in which the catalytic WCPPC redox motif was found. This redox active protein is present solely in the representatives of the Kinetoplastida. TXN is localized in cytosol (TXN-I), as well as in the mitochondrial and endoplasmic reticulum compartments (TXN-II). The latter isoform is present as an integral membrane protein, with its catalytic site facing to cytosol [102]. Reduction of both TXN-I and TXN-II depends on TSH, and electrons are then transferred to a diversity of acceptors, such as GSSG, Trx, GPx and tryparedoxin peroxidase (TXNPx) [103]. However, these proteins are less efficient in the detoxification of ROS as compared with GPx [104].

##### Plasmoredoxin (Plrx)

This redox-active protein represents an interesting variant of the Trx superfamily. With a higher molecular weight (21.4 kDa) as compared with the typical members of the superfamily [105], Plasmoredoxin is typical of the Apicomplexa and can be reduced by either TXN or Grx, although with a very low efficiency. GSH and Trx can also act as electron donors to Plrx [106].

## 3. Peroxidases

### 3.1. General Features of Peroxidases

The major antioxidant enzymes involved in the reduction of H_2_O_2_ are CAT and the selenocysteine-dependent glutathione peroxidase (Sec-GPx). Its catalytic efficiency is in the same range (*k*_cat_/K_m_ 10^7–8^ M^−1^s^−1^) [26,107,108]. In contrast, the corresponding value for peroxiredoxins (Prx) is far below (10^4–5^ M^−1^s^−1^) [26,109,110]. However, values of 10^7^ M^−1^s^−1^ for some Prxs have been reported [26]. As was noted above, Prx as well as the GPx, are the enzymes responsible for H_2_O_2_ reduction. The catalytic ability of such enzymes depends on the presence of either cysteine or selenocysteine in the active site.

In its free state in solution, the pKa value of the -SH group of Cys is about 8.4. By contrast, the corresponding value for the selenol group (-SeH) of selenocysteine (Sec) is 5.2. Thus, the reactivity of the latter as nucleophile is higher as compared with that of Cys. However, it has been demonstrated that in the active site of some enzymes (e.g., Prx) the pKa value of the reactive Cys can reach a value of ~5.0 [111] thus increasing its nucleophilic character. In this sense, the second order rate constant for free cysteine in the disposal of H_2_O_2_ has a value of 18–26 M^−1^s^−1^ [112]. In comparison, the corresponding value for the reactive Cys in Prx is 10^5^ M^−1^s^−1^ [26,109], while for Sec in GPx the second order rate constant increase up to 4 × 10^7^ M^−1^s^−1^ [108]. Such differential reactivity was corroborated by site-directed mutagenesis. Thus, in a mutant of GPx in which Sec was replaced by Cys [113], the activity of the enzyme, with H_2_O_2_ as the substrate, was decreased by three orders of magnitude as compared with the wild-type enzyme.

### 3.2. Characteristics of Thiol-Dependent Peroxidases

#### 3.2.1. Glutathione Peroxidase (GPx)

GPx are generally homotetrameric proteins constituted by subunits of 19 to 25 kDa. Its activity is dependent on either Cys (GPx5 and GPx6) or Sec (GPx1 to GPx4) and are capable to reduce H_2_O_2_ and organic hydroperoxides (except lipid hydroperoxides) using GSH as reducing substrate. The variant GPx4 (see below) represents an atypical case, because it is capable to reduce lipid hydroperoxides using a variety of reductants, in addition to GSH [114]. The common denominator of the GPx family are highly conserved residues such as Try, Asp and Glu near the catalytic site [108]. The multiple variants of GPx (EC 1.11.1.9) in mammals, can be grouped into the following six families:

(i) GPx1 (cytosolic). Represents the typical GPx which is widely distributed in tissues. The enzyme can metabolize hydrogen peroxide and various organic peroxides but cannot metabolize fatty acid hydroperoxides present in phospholipids [115].
(ii) GPx2 (gastrointestinal). This isoform is similar to GPx1 in terms of substrate specificity, and is present in liver and large intestine but not in other organs [116].
(iii) GPx3 (plasmatic). It is a glycoprotein with reductase activity similar to that of GPx1; however, millimolar concentrations of GSH for activity are needed. Trx and Grx can also act as electron donors for GPx3 [117]. The enzyme is present mainly in kidney [118].
(iv) GPx4 (Phospholipid hydroperoxide GPx). This variant of GPx is a monomeric protein that react mainly with phospholipid hydroperoxides as substrate, and is capable to accept a wide range of reducing substrates, including GSH [119].
(v) GPx5 (epididymis). A low activity epididymis-specific GPx, its activity with H_2_O_2_ or organic peroxides is less than 0.1% of that of GPx1 [120].
(vi) GPx6 (odourant metabolism). It was found in the Bowman´s gland of the olfactory system [121].

**Figure 4:**
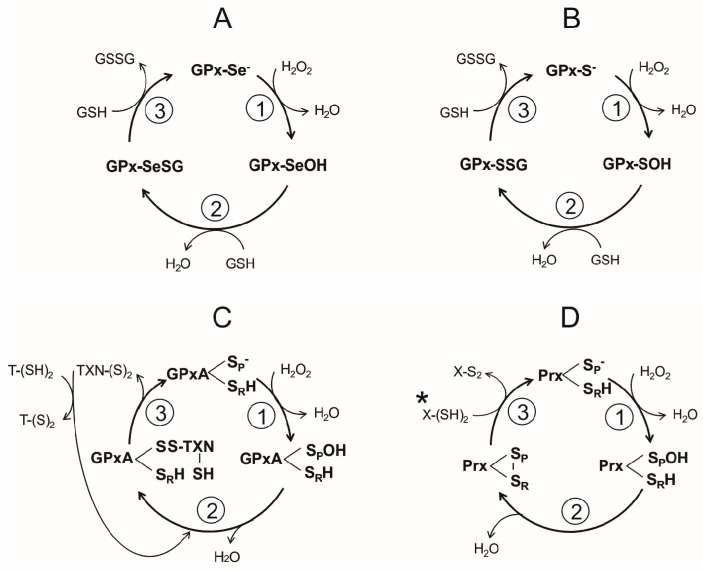
General catalytic cycles involved in H_2_O_2_ reduction by either thiol- or selenol-dependent peroxidases in invertebrate parasites. (**A**) Sec-dependent Glutathione Peroxidase; (**B**) Cys-dependent Glutathione Peroxidase; (**C**) dithiol Glutathione Peroxidase-like from Kinetoplastida. Although structurally similar to GPx, the peroxidase from Kinetoplastida is catalytically similar to the tryparedoxin-dependent peroxiredoxin. X-(SH)_2_ stand for a dithiol protein from the thioredoxin family (**D**) dithiol peroxiredoxin.

The catalytic cycle of the Sec-dependent GPx can be summarized in the following three steps (Figure 4A):

(i) Reduction of the peroxide. In the first step of the reaction, a nucleophilic attack on the peroxide bond by the reactive selenolate (-Se^−^) leads to the formation of a selenenic acid intermediary (GPx-SeOH) and the release of the first water molecule. Such intermediary appears to be a common feature in the catalytic cycle of all the Sec-dependent GPx.
(ii) Formation of the covalent adducts selenocysteine-glutathione (GPx-SeSG). In this step, a GSH molecule reacts with the selenenic acid intermediary, producing the second water molecule and a mixed selenil-sulfide covalent intermediary.
(iii) Regeneration of selenolate. In the third step of the reaction, a second GSH molecule reacts with the mixed selenil-sulfide intermediate, leading to the regeneration of the initial selenolate state of the enzyme. During this last step a GSSG molecule is produced.

A reaction sequence essentially identical is followed by the Cys-dependent GPx, differing only in the chemical nature of the intermediates (Figure 4B). Thus, a sulphenic acid is produced in the first step, followed by the formation of a mixed disulfide (Figure 4B,C). In the final stage of the reaction, the -S^−^ is regenerated.

**Figure 5:**
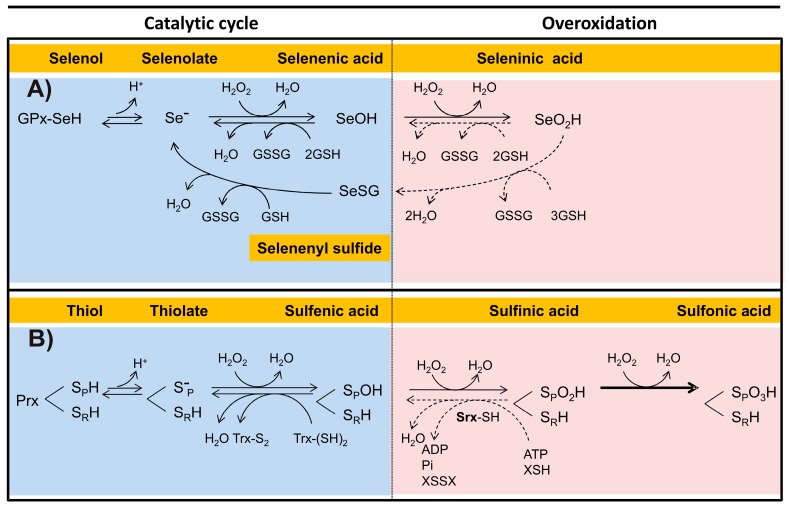
Mechanisms involved in the overoxidation of the catalytic Sec or Cys in representative enzymes. Both the catalytic and the overoxidative pathways in a Sec-dependent Glutathione Peroxidase (**A**), and in a Cys-dependent peroxiredoxin (**B**) are detailed. The different oxidation states for both Sec and Cys are shown. Reversible reactions in the overoxidative pathways are indicated by dotted lines. Abbreviations are as follows: sulfiredoxin (Srx); reduced thiol-dependent substrate (XSH); disulfide form of a thiol-dependent substrate (XSSX).

An alternate reaction pathway is available to the Sec-dependent GPx, in which the selenenic acid intermediate is capable to reduce a second H_2_O_2_ molecule in the same catalytic cycle. However, as a result of such reaction, a seleninic acid oxidation state (GPx-SeO_2_H) is produced, representing an over oxidized inactive state of the catalytic selenocysteine. The regeneration of the -Se^−^ will be dependent on GSH, requiring a total of four of such molecules. This latter process can take place through either of two alternate routes (Figure 5A). In the first option, two GSH molecules are required in the reduction of SeO_2_H into SeOH. Then, two additional GSH molecules leads from SeOH to the Se^−^ state of the catalytic Sec residue of the enzyme. The alternate regeneration pathway involves the formation of a selenyl-sulfide mixed intermediate, requiring three GSH molecules. In the second stage of the process, an additional GSH molecule allows the final reduction to Se^−^. In either of the two alternate pathways, two GSSG molecules are produced.

#### 3.2.2. Peroxiredoxin (Prx)

Peroxiredoxins are a ubiquitous group of peroxidases, which are present in all the living organisms. They are characterized by the presence of either one (1-Cys-Prx) or two (2-Cys-Prx) catalytically active cysteine residues [109]. The following classification of Prxs is based on the PeroxiRedoxin Classification Index (PREX) (http://csb.wfu.edu/prex), which considers six subfamilies (Prx1, Prx5, Prx6, PrxQ, Tpx, and AhpE):

(i) Prx1 (2-Cys Prx) are the typical Prx. They are dimeric proteins but are capable to aggregate into decamers. They are well represented in the living world, being the major form of Prx. The eukaryotic variant is prone to over oxidation and has a higher activity with H_2_O_2_ over organic peroxides [26]. In humans it is represented by the isoforms PrxI, PrxII, PrxIII, and PrxIV [122].
(ii) Prx6 (1-Cys Prx) are mainly dimeric and, in some cases, polymeric enzymes [123] and are distributed in Archaea, Eubacteria, and Eukaryotes [124].
(iii) Prx5 (1-Cys Prx and 2-Cys Prx) are dimeric proteins and have a wide distribution, being present in bacteria, fungi, plants and mammals [124].
(iv) PrxQ (1-Cys Prx and 2-Cys Prx) is present in Archaea, Eubacteria, plants and some Eukaryotes, but it is absent from mammals [124]; some members are monomeric [125].
(v) TPx (2-Cys Prx), also called thioredoxin peroxidase. They are found in bacteria [26].
(vi) AhpE (1-Cys Prx and 2-Cys Prx). This variant of Prx is present in aerobic gram-positive bacteria of the order Actinomycetales [124].

A feature that distinguishes certain Prx subgroups involves the contact interfaces between subunits in dimers. Based on this characteristic, two kinds of dimeric Prx are recognized: (i) A-type dimers, where the major contact between monomers is through α-helicoidal segments as exemplified by Prx1, Prx6 and AhpE; (ii) B-type dimers, where the interaction is through β-sheets as in Prx5, PrxQ and TPx. Dimeric Prx1 and Prx6 can aggregate to form decamers or dodecamers. However, the biological significance of these oligomeric states is unclear [126].

Unlike GPxs, organic peroxides and peroxinitrite (ONOO^−^) are better substrates for Prxs [26]. Further, in some cases they are capable to use a variety of reducing substrates [127]; this latter possibility will be dependent on the organism.

In the catalytic cycle of the dimeric typical Prxs two essential cysteine residues are involved: the peroxidatic cysteine (S_P_H) and the resolving cysteine (S_R_H), which are located each in the neighbor subunits. Generally, the Prxs conserve an active-site Arg, which act by lowering the p*K*_a_ of the S_P_H and stabilize its thiolate form [109], so that at physiological pH S_P_H is found predominantly in the ionized state (S_P_^−^), while S_R_H remains in its non-dissociated state. Like GPxs, the catalytic cycle of Prx can summarize into three stages (Figure 4D):

(i) Peroxide reduction. In the first step, the nucleophilic attack by the S_p_H on the O-O covalent bond of H_2_O_2_ leads to the formation of the intermediary sulphenic acid state of the peroxidatic cysteine (S_p_OH) and the release of the first water molecule. Such intermediary is apparently shared between various Prxs.
(ii) Formation of the disulfide bond. The formation of the second water molecule involves the oxidation of the catalytic cysteine residues into an intramolecular disulfide bond, as result of the nucleophilic attack of the intermediary sulphenic acid by S_R_H.
(iii) Reduction of the intermediary disulfide bond of Prx. This last step results in the regeneration of both S_P_^−^ and S_R_H catalytic residues and is dependent on a reducing agent, typically a redoxin protein in which a conserved CXXC redox motif is present.

The above chemical mechanism is shared by a variant of the two-cysteine dependent Prxs, in which both S_P_H and S_R_H are localized in the same subunit. By contrast, in the one-cysteine dependent Prxs type, the S_R_H residue is absent and the regeneration of the initial thiolate depends on the presence of a low molecular weight thiol [109].

Like selenenic acid, the sulphenic acid intermediate of the 2-Cys Prxs is capable of reducing a second H_2_O_2_ molecule, leading to the formation of the sulphinic acid form (S_P_SO_2_H), which represents an over-oxidized state (oxidation number + 4) of sulfur in Cys. In some Prxs, its formation can be reverted. Thus, in the reduction of the sulphinic acid intermediate of the 2-Cys Prx a specific protein, named sulfiredoxin (Srx), is involved. It is dependent on both ATP and a reducing substrate, either GSH or Trx [128,129] (Figure 5B). Although the over-oxidized state can also be reverted by sestrin, information about its properties is scarce [130].

In the presence of a constant oxidative stress the sulphinic acid intermediate of Prxs has the potential to reduce a third H_2_O_2_ molecule, leading to the formation of the sulphonic acid (S_P_SO_3_H) oxidized state of the sulfur in peroxidatic cysteine. Such over oxidized state of cysteine, in which its oxidation number has been increased up to +6, leads to an irreversible inactivation of the enzyme [128,129] (Figure 5B). To date there are no reports about an over oxidized state of the catalytic cysteine residue of GPx, although such event is potentially feasible. As regard the catalytic selenocysteine in Sec-GPx, an over-oxidation of such residue is not possible.

## 4. Disulfide Reductases

### 4.1. Why Do Parasites Need Reductases?

In the previous sections, the role that different thiol-compounds play in the protection against oxidative stress was discussed. As result of their participation in the defense mechanisms, the oxidized state of protective molecules such as glutathione and thioredoxin is produced. To maintain a functional pool of such compounds, their reduced state must be regenerated. Hence, the participation of disulfide reductases is critical for cell redox homeostasis. In this section, the mechanistic details of this group of enzymes is discussed.

### 4.2. Characteristics of Thiol-Dependent Reductases

#### 4.2.1. Glutathione Reductase (GR) and Trypanothione Reductase (TryR)

Glutathione reductase (GR) is widely distributed in living organisms, albeit it is not of universal occurrence [65]. By contrast, the presence of trypanothione reductase (TryR) is restricted to the representatives of the phylum Kinetoplastida [131]. Both enzymes are dependent on NADPH as electron donor.

Unlike its homologous thioredoxin reductase (TrxR), GR has remained conservative along the evolutionary history [132]. Thus, the sequence comparison of GR from a diversity of species, reveals a high identity [65]. In this sense, the enzyme from organisms as different as *E. coli* and human, share an identity of 50% [132]. Although the disulfide substrate of both GR and TryR share a glutathione disulfide moiety, the presence of a spermidine bridge in trypanothione makes it an intramolecular disulfide [72].

**Figure 6:**
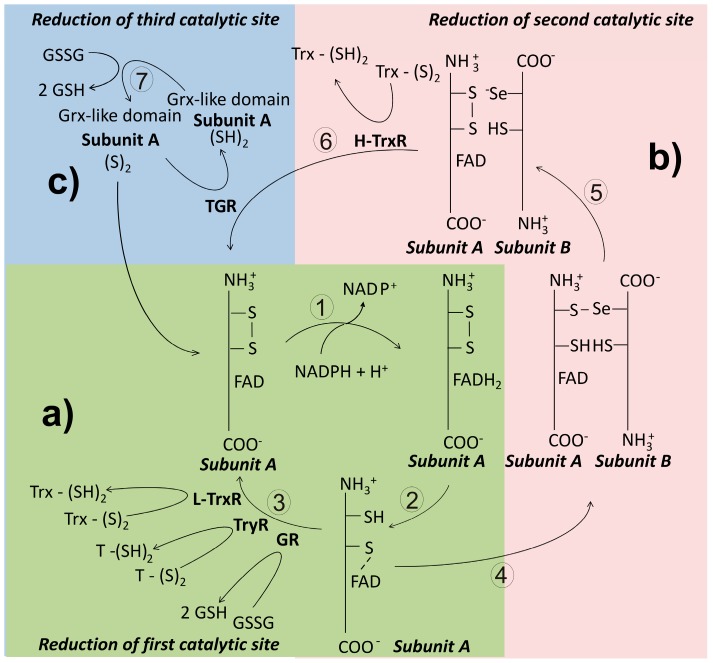
General catalytic mechanism of disulfide reductase enzymes in invertebrate parasites. In panel (**a**) a simplified pathway for electron transfer from NADPH to a disulfide substrate is shown. This route is present in enzymes such as glutathione reductase (GR), trypanothione reductase (TryR), and the low-molecular weight thioredoxin reductase (L-TrxR). Panel (**b**) shows the additional reactions involved in the disulfide reduction followed by the high-molecular weight thioredoxin-reductase (H-TrxR). In panel (**c**) the additional pathway for electron transfer found in thioredoxin-glutathione reductase (TGR) is shown.

From both the structural and the functional viewpoint, GR and TryR are closely related (Figure 6). The binding site for the disulfide substrate is constituted by residues contribution of four α-helix of one subunit and the C-terminal end of the neighbour subunit [133]. However, due to the differences between GSSG and oxidized trypanothione (T(S)_2_), the electrostatic environment in the corresponding binding site of both enzymes is different [131]. In GR, the presence of positive residues allows binding of the negatively charged GSSG, while in TryR negative residues, as well a broad cavity, render possible binding of the positively charged T(S)_2_ [134]. However, it is interesting to note that by changing just two amino acid residues in GR, binding of T(S)_2_ becomes possible, thus demonstrating the close relationship between both enzymes [135].

#### 4.2.2. Thioredoxin Reductase (TrxR)

Thioredoxin reductase (TrxR) is a flavoenzyme involved in the reduction of the disulfide bond of thioredoxin. In most organisms, the presence of both Trx and TrxR is essential in the production of reduced ribonucleotides [38]. Along its evolutionary history, TrxR has diverged leading to the apparition of three variants of the enzyme [136]. Thus, in prokaryotes and plants, TrxR is present as a dimeric protein constituted by subunits of about 35 kDa whose subunits lacks the interface domain [137]. This isoform is named as the low molecular weight TrxR (L-TrxR). By contrast, in animals the enzyme exists predominantly as a dimeric protein with subunits of about 55 kDa, and has been named as the high molecular weight TrxR (H-TrxR). Unlike the prokaryotic variant, in the animal H-TrxR an additional redox active center is located at the C-end of the polypeptide chain [7]. In this site either a dithiol/disulfide [138] or a selenol-thiol/selenylsulfide [139] can be present. Mammalian TrxR is critically dependent on Sec for activity [140]. Finally, in a variant of the H-TrxR, a glutaredoxin-like domain is present at the N-terminal end of the subunit, making it a multifunctional enzyme. This isoform of the H-TrxR retains the C-terminal redox center found in others H-TrxR. Based on its ability to use either GSSG or Trx-S_2_ as substrates, the enzyme has been named thioredoxin-glutathione reductase (TGR) [141].

##### Low Molecular Weight Thioredoxin Reductase (L-TrxR)

As noted above, this variant of TrxR is distributed in bacteria, plants, fungi and some Protista [142]. Unlike its high molecular weight counterpart, the L-TrxR is highly specific in the reduction of its cognate Trx [137]. It has been proposed that both L-TrxR and H-TrxR arose from an ancestral nucleotide binding protein [136], so that its TrxR activity was the result of convergent evolution.

In the catalytic mechanism of the L-TrxR a great conformational change of the enzyme is required, such that the NADPH binding domain rotates about 67° related to the FAD binding domain after binding of NADPH [136]. Electrons are then transferred to FAD and from here to the catalytic CXXC redox motif (Figure 6). This latter process allows the exposure of the Trx binding site, leading to binding and reduction of Trx-S_2_. From the crystallographic evidence with the Trx-TrxR complex from *Saccharomyces cerevisiae*, it has been shown that in the recognition of the disulfide substrate, electrostatic interactions are involved [137].

##### High Molecular Weight Thioredoxin Reductase (H-TrxR)

This variant of TrxR is characteristic of animals, where is present in both the cytosolic and the mitochondrial compartments [7]. As noted above, the enzyme activity of the high molecular weight variant of thioredoxin reductase (H-TrxR) is critically dependent on the presence of an additional redox active center located at the C-terminal end of the subunit (Figure 6) [143], where either a dithiol/disulfide or a selenol-thiol/selenylsulfide is found. In mammals, the consensus sequence Gly-Cys-Sec-Gly (GCUG) is present in such redox center [7]. As described above for both GR and TryR, the reducing equivalents coming from NADPH pass sequentially through the FAD prosthetic group and the N-terminal disulfide [144]. Then, they are transfer to the C-terminal redox center of the adjacent subunit. The low pKa value of the active Sec residue (about 5) [139] makes it an excellent nucleophilic reagent [145]. Thus, following binding of the substrate Trx, an intermediary enzyme-substrate mixed selenyl/sulfide is formed as result of the nucleophilic attack of the reactive -Se^−^ on the disulfide bond of Trx [146]. Finally, the adjacent Cys of the C-terminal redox motif leads to release of reduced Trx through a nucleophilic attack. Unlike the homologous GR and TryR, during the catalytic cycle of H-TrxR the enzyme fluctuates between the two- and the four-electrons reduced state [147]. Recently, the crystallization and resolution of the three-dimensional structure of both the human [99] and the *P. falciparum* TrxR-Trx complex was reported [148].

An interesting observation on the Sec-dependent H-TrxR concerns to its ability to reduce Trx substrates from a diversity of sources, in addition to its cognate [144,149]. To explain this lack of specificity for its disulfide substrate, it has been proposed that the structural and electrostatic features of the Trx binding site were conserved through evolution [99,150,151]. In this sense, the environment surrounding α-helix 3 of eukaryotic Trx shows a high density of negatively charged residues, which is complementary to a positively charged α-helix at the binding site on H-TrxR [151].

##### Thioredoxin-Glutathione Reductase (TGR)

This variant of the H-TrxR represents an example of a chimera protein in which a Grx-like domain was appended to the N-terminal end of the animal TrxR [141]. Such domain combination allows TGR to reduce both Trx-S_2_ and GSSG, as well as catalyze deglutathionylation reactions [152]. Hence, TGR is a multifunctional enzyme. As compared with rat H-TrxR, TGR shows an identity of 57% [153]. The Grx-like domain, in which either a monothiol [154] or a dithiol [155] redox motif can be present, adds an additional redox-active center to the enzyme (Figure 6). Interestingly, the presence of the Grx-like domain is not required for Trx-S_2_ reduction [11]. Like mammalian H-TrxR, TGR depends on a Sec residue for its disulfide reductase activities [155,156]. The enzyme was described first in mouse testes [141], where is expressed at significant levels only during spermatid maturation [157]. In parasite flatworms, TGR is the only disulfide reductase present (see below). Results from both kinetic and crystallographic studies with *S. mansoni* TGR have revealed that the mechanistic details for Trx-S_2_ reduction are essentially identical to that of the mammalian H-TrxR [153]. As regard reduction of GSSG by TGR, the presence of the Grx-like domain is critical, although the mechanistic details of electron transfer remain to be elucidated.

### 4.3. Reductase-Independent Substrate Reduction

Although the presence of specific disulfide reductases in the antioxidant defense systems of the parasites is critical for its survival, in vivo a redox reaction involving a reduced and an oxidized compound is also feasible. Such possibility depends on both similar redox potential of the participating compounds and an adequate reduced/oxidized concentration ratio, as predicted by the Nernst Equation [61]. In this sense, transgenic rats lacking H-TrxR are fully normal, suggesting the GSH system is capable to compensate the absence of H-TrxR [158]. In *P. falciparum* GSH, as well as Grx and TXN, can reduce directly Plrx [106]. On the other hand, in non-altered biological systems there are examples in which the non-enzymatic reduction of a disulfide can compensate the loss of its specific disulfide reductase. Thus, in insects such as *Drosophila melanogaster* GR is absent, and the Trx/H-TrxR system is involved in the reduction of GSSG [138]. In the genus *Trypanosoma* a direct electron transfer between TSH and TXN has been noted, as result of very similar redox potential and in vivo concentrations [159]. This latter fact allows a quickly response by *Trypanosoma* to minor changes in the redox status of the parasite.

## 5. Architecture of Thiol-Dependent Antioxidant Systems in Invertebrate Parasites

### 5.1. Protista Parasites

#### 5.1.1. Phylum Amoebozoa

*Entamoeba* is a typical representative of this group of unicellular parasites. It lives in microaerophilic environments where it is exposed to very low oxygen partial pressures (5 mm Hg) and their metabolism is essentially anaerobic. However, after infection of a host it becomes exposed to a higher oxygen tension. Hence, the presence of an antioxidant defense system is critical for survival. In *Entamoeba* the GSH-dependent antioxidant system and the enzymes involved in glutathione biosynthesis are absent, albeit the presence of GSH at micromolar concentrations (20 μM) has been reported [160]. Instead, Cys represent the main low molecular weight antioxidant, reaching concentrations of about 200 μM [161]. Although *Entamoeba* is capable of synthesizing Cys by the de novo pathway [43], when it is grown in an axenic medium lacking Cys their endogenous production of ROS increases up to four times. Such a raise in ROS content can be prevent by adding Cys to the culture medium, suggesting the biosynthetic ability of the parasite for cysteine is limited [48]. Under this latter condition, an increase in Cys-transporters of the major facilitator superfamily (MFS 2) has been reported [162]. The oxidized Cys incorporated or oxidized by ROS can be directly reduced by atypical NADPH-dependent oxidoreductases (EhNO-1 and 2 in *E. histolytica*) [163]. In the presence of radical compounds, Cys can be derived into S-nitrocysteine (Cys-NO), being reduced through the Trx antioxidant system [43].

**Figure 7:**
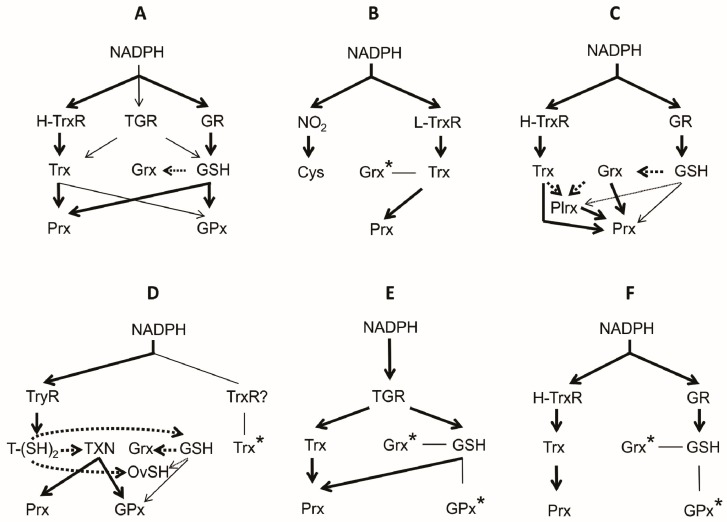
Architecture of the thiol-based antioxidant systems in animals. The organization of the antioxidant redox system present in the phylum Vertebrata (**A**); and in the parasite representatives of the phyla Amoebozoa (**B**); Apicomplexa (**C**); Kinetoplastida (**D**); Platyhelminthes (**E**); and Nematoda (**F**) phyla are shown. In each case, the predominant and the minor electron flow is indicated as a continuous thick arrow and a continuous thin arrow, respectively. Potential electron transfer reactions involving only redox substrates is represented by a dark (highly favoured) or light (poorly favoured) dotted arrow. From bioinformatic analysis, the presence of some components (*) is proposed. The debatable presence of a TrxR in the representatives of Kinetoplastida is indicated by a question mark (?).

The enzymatic machinery to contend with the oxidative stress depends on both Prx and Trx. In *E. histolytica* a total of three Prxs have been reported, of which Prx 1 is localized in the cytosolic compartment. This isoform is over-regulated in the trophozoite stage under conditions of oxidative stress [164]. Due to the absence of GSH, the reducing equivalents needed for the Prx function are provided by the Trx-redox system [165]. Both Trx and TrxR are present, the latter represented by the L-TrxR variant [166]. Interestingly, the L-TrxR is capable to receive electrons from either NADH or NADPH [167]. On the other hand, in the related *Giardia labia* the presence of two Prxs has been reported, although information about its biochemical properties is lacking [168]. A summary of the architecture of the thiol dependent antioxidant system in the group of Amebozoa is shown in Figure 7B.

The most outstanding feature of the antioxidant system in the Amoebozoa lies in the mechanism to contend with H_2_O_2_. In this process two specific oxidoreductases are involved: pyruvate-ferredoxin oxidoreductase (PFOR) [169,170], and the NADPH-dependent rubredoxin reductase (NROR) [171], which act in concert. PFOR participates in the oxidative decarboxylation of pyruvate transferring electrons to ferredoxin and then to O_2_, leading to the formation of H_2_O_2_. Interestingly, the activity of the enzyme allows the activation of metronidazole [172]. PFOR is located at a subcellular membrane although its exact position depends on the species. In *Trichomona vaginalis* the enzyme is find at the hydrogenosome membrane [173]. As to NROR, the enzyme transfer reducing equivalents from NADPH to H_2_O_2_, using either rubredoxin or rubrerythrin as intermediates [43]. As result, H_2_O_2_ is reduced to H_2_O. In *Giardia intestinalis* both enzyme activities are critical in the maintenance of its highly reducing intracellular environment. Both PFOR and NROR are expressed in the presence of O_2_ [174].

#### 5.1.2. Phylum Apicomplexa

Although this group of parasites includes about 5500 species, *P. falciparum*, the cause of malaria in humans, is by far the most conspicuous and important member. *P. falciparum* is a facultative organism whose life cycle involves an intracellular stage inside the human erythrocyte. In this compartment, the parasite is exposed to a high oxygen tension and hence to the deleterious effect of ROS. However, in *P. falciparum* both the glutathione- (GR, GSH and Grx) and the thioredoxin- (TrxR, Trx, Prx) dependent antioxidant systems are present (Figure 7C) [35], which are distributed in all its subcellular compartments. Even in the parasitophorous vacuole surrounding the parasite, the presence of a Trx2 has been reported [175]. Hence, *Plasmodium* is well equipped with an antioxidant machinery to contend with ROS.

When the parasite is located inside the red blood cell of its host, the GSH concentration oscillates between 0.4 mM to 2.3 mM [105]. During the invasive process of the erythrocyte, GSH of *P. falciparum* become oxidized, leading to its accumulation as GSSG. The latter is then secreted, resulting in depletion of the intracellular pool of GSH. To restore its normal concentration values, an active glutathione biosynthesis by the parasite is required [176]. Thus, in this parasite the GSH-biosynthetic pathway is of a major importance for its survival [177].

In *P. falciparum* both a low molecular weight (12.2. kDa) 1-Cys, as well as a high molecular weight (20 kDa) 2-Cys variants of Grx are present [178], which have been related to the high glutathionylation activity to protect proteins from oxidation [41]. In this process Trx is also involved. In addition to the above antioxidant systems, in *P. falciparum* another redox active protein is present. It has been named plasmoredoxin (Plx), and it is exclusive to the genus *Plasmodium* [105]. The protein retains the typical Trx fold and shows the sequence WCKYC at its redox active motif. Plx is involved in the reduction of RR and in protein deglutathionylation, like Grx and Trx [179].

Due to the absence of CAT and GPx, in *P. falciparum* the redox defense mechanism against peroxides depends on Prxs. In this parasite, five isoforms of Prx have been reported: cytosolic Prx1a [180], mitochondrial Prx1m [181], Apicoplast Prx5, as well as Prx6 and PrxQ [26,180]. In that follows, the main characteristics of this group of enzymes are briefly described. Prx1a is the most studied isoform. The enzyme is present through all the life cycle of the parasite [180] and is capable to reduce H_2_O_2_, ONOO^−^, cumene hydroperoxide (CHP), and t-butyl hydroperoxide (tBuOOH) [182,183,184]. For its activity, Prx1a depends on either Trx or Plrx [105] (Figure 4C). The Prx1m isoform (2-Cys Prx) is expressed in the schizont and the trophozoite stages of the life cycle [181]. Both Trx-1 and Trx-2 are involved in the regeneration of the reduced form of this Prx variant [105]. Prx5, also known as antioxidant protein (AOP), shows a high identity with GPx [185]. Based on this observation, Prx5 was included inside the GPx family. However, since the enzyme shows higher activity with Trx as compared with GSH, it was included into the Prx family of enzymes. Prx5 is more efficient in the reduction of tBuOOH and phospholipid peroxides as compared with H_2_O_2_ and CHP, and endogenous Grx is a better reducing substrate of Prx5 than Trx [178]. Although the transcript of Prx5 is present in all the life cycle stages inside the erythrocyte, its concentration is maximal in the trophozoite stage. The enzyme is located at the Apicoplast [186,187].

Prx6 is capable of reducing both H_2_O_2_ and tBuOOH with either Trx or Grx as electron donors. The potential use of GSH by the enzyme has been not elucidated [188]. Finally, PrxQ (nPrx) is a nuclear Prx with preference of Grx over Trx in the reduction of both H_2_O_2_ and CHP [188].

GR and TrxR are present in Apicomplexa, as deduced from the presence of a single copy of each gene. In both cases, the diverse enzyme isoforms present in different cellular compartments of these Protista are generated by alternative translation initiation [175]. GR from *P. falciparum* has been extensively studied [189,190]. The enzyme shows an identity of 35% as compared with its human homologue [189], and the amino acid residues involved in the catalytic cycle are identical in both cases. However, differences at the glutathione-binding pocket and the intersubunit contact area between the human and the parasite enzymes are present. In human GR, an arginine residue (R347) is involved in the binding of GSSG through a salt bridge [191]. By contrast, in the *Plasmodium* enzyme the equivalent residue is a glutamate (E374). As to the interface area, the cysteine residue (C90) participating in an intersubunit disulfide bond in human GR is lacking in the *Plasmodium* enzyme [189].

As regard TrxR, in *P. falciparum* the enzyme is represented by an H-TrxR in which Sec has been replaced for Cys at the C-terminal redox center [192]. The catalytic mechanism of the enzyme has been intensively studied [193,194]. In the reduction of Trx, the reducing equivalents from NADPH follows the same pathway described for H-TrxR. Recently, the crystal structure of the Trx-TrxR binary complex has been resolved [148]. The enzyme-substrate interactions are essentially identical to that observed in the corresponding complex from human [99].

Finally, it is worth noting that in the Apicomplexa an interesting variant of the Trx superfamily was found. The protein, named plasmoredoxin (Plrx), can be reduced by either TXN or Grx. Interestingly, in a knock out mutant of *P. berghei* lack of Plrx no difference in survival was found when compared with the wild type, suggesting Plrx is not essential for the infective process [99].

#### 5.1.3. Phylum Kinetoplastida

The phylum Kinetoplastida consists of a large group of parasitic flagellated Protista causing infections in humans and animals. The most important infectious diseases in man caused by this kind of unicellular parasites are sleeping sickness and Chagas diseases (*Trypanosoma* species), as well as leishmaniasis (*Leishmania*). The poorest regions of developing countries in tropical and subtropical areas of the world are the more affected by these parasites [195].

Members of the Kinetoplastida have adapted to a diversity of environmental conditions, and hence they are exposed to different oxygen tensions. Their life cycle involves different intermediary hosts and can be found both intra- or extracellularly. Interestingly, in these organisms a diversity of sulfhydryl compounds are present. The main antioxidant redox system of these parasites is based on the TSH redox system [79,196], which include a specific disulfide reductase as well as a Trx-like protein called tryparedoxin (TXN) [197,198]. In these parasites, the concentration of TSH is in the range 0.2 mM to 1.5 mM [17].

Although GR and TrxR are absent (Figure 7D), the existence of both GSH and Trx in these parasites have been reported [199,200,201]. It is worth to note that in some species of the Kinetoplastida GSH is present at similar concentrations to that of TSH [202]. Due to the higher reducing ability of TSH as compared with GSH, the latter remains mainly in its reduced state (Figure 7D). In the Kinetoplastida two metabolic routes for Cys biosynthesis, as well as for GSH, are present [36]. Some species can synthesize TSH or spermidine, or incorporate these from the medium [203]. In some species such as *Trypanosoma cruzi*, a requirement for exogenous polyamines have been reported [204].

In this kind of parasites, the Trx-like TXN act as electron donor to TSH, which transfers them to target proteins such as Prx [205], RR [37], and MSR [95]. The levels of TXN depends on the specific stage of the life cycle of the parasite, being particularly abundant in the trypomastigote stage [206]. By using interference RNA in *T. cruzi* the importance of TXN in the parasite survival has been demonstrated [207].

In addition to the TSH redox system, in the Kinetoplastida the existence of the low molecular weight redox proteins Grx and Trx have been demonstrated. For Grx, both the monothiol and the dithiol variants are present [208]. In this sense, in *T. brucei* two isoforms of the two-cysteine variant of Grx have been reported: 2-Cys-Grx 1 and 2-Cys-Grx 2. The former is located at the cytosolic compartment while the latter is find at the mitochondrial intermembrane space, at concentrations of 2 μM and 0.2 μM, respectively. They are expressed constitutively both in the procyclic and the blood stages of the parasite, where act as electron donors for RR [91]. Experimental evidence has revealed that the presence of the 2-Cys-Grx 2 is critical for the parasite survival, affecting the growth of the procyclic form of the life cycle. In contrast, the deletion of the 2-Cys-Grx 1 variant has no effect [91]. As to Trx, its presence in all the stages of the life cycle of *T. brucei* has been reported, where appear to be involved in the reduction of RR and Prx. The absence of this redox protein in a null mutant result in a slower growth rate as compared with the wild type [84]. However, it has been proposed in the Kinetoplastida Trx has no major physiological relevance [196].

In these parasites, the antioxidant enzyme machinery to contend with H_2_O_2_ consists of a dithiol dependent Prx (2-Cys Prx) as well as a glutathione peroxidase-like named tryparedoxin peroxidase (GPx, Cys-GPx and Px). In the case of GPxA from *T. brucei*, residues Cys47, Gln82, and Trp137 are functionally equivalent to Sec, Gln, and Trp, respectively, of the catalytic triad of the classical mammalian GPx [134]. GPxA is a monomeric protein whose catalytic activity depends on a Cys residue. Like mammalian GPx4, GPxA from *T. brucei* has preferential activity toward phospholipid hydroperoxides [134]. In the trypanosomatids, the enzyme contains a resolving cysteine (C_R_) for its catalysis and is reduced by redoxins [108]. GSH is a poor electron donor [75]; however, the reducing activity of GPxA increases with TXN [108]. Both 2-Cys Prx as well as GPxA displays tryparedoxin peroxidase activity (TXNPx) [75] (Figure 4C,D). It is worth to note that both H_2_O_2_ and ONOO^−^ are the main substrates of the 2-Cys Prx, while GPxA acts on lipid hydroperoxides [134]. As to Prxs, in the representatives of the Kinetoplastida four isoforms (PxI, PxII, PxIII, and Px IV) have been found [134]. The catalytic efficiency of such enzymes is in the same range as compared with the 2-Cys Prxs, although minor in comparison with the Sec-GPx [134]. Lastly, in this kind of parasites a third kind of peroxidase, named ascorbate peroxidase (APx), has been reported. The enzyme is capable to accept reducing equivalents from TXN for peroxide reduction [209,210]. The three kinds of peroxidases above discussed are dependent on both the TSH and the TXN redox systems for activity.

As consequence of their reductant role and electron-donator of Prxs and GPx, the disulfide form of TSH is produced. The latter is reduced by TryR, the unique NADPH-dependent disulfide reductase, exclusive of the Kinetoplastida. Hence, in this kind of parasites TryR is critical for its reproduction and survival [211].

Finally, in this group of unicellular parasites the presence of OSH, a compound typical of marine invertebrates, has been documented [76]. OSH is present through all the stages of the life cycle at concentrations ranging from 0.1 mM to 1 mM. In the amastigote stage of *Leishmania* the variant A of OSH is present at a concentration which is similar to that of TSH [212]. However, in other representatives of the Kinetoplastida the OSH concentration is variable during the different stages of the biological cycle [77].

### 5.2. Metazoan Parasites

#### 5.2.1. Phylum Platyhelminthes

The organisms included in this set of acoelomated metazoa are characterized by the lack of both respiratory and circulatory systems. The parasitic species includes the known flukes (Class Trematoda) and tapeworms (Class Cestoda).

In this group of animal parasites GSH, as well as Trx and Grx are of great importance in the redox metabolism. Trx is present in both tapeworms and flukes. In the larval stage of *Echinococcus granulosus* two isoforms of Trx has been reported [213], which are located at the cytosolic and the mitochondrial compartments. In the metacestode (cysticerci) stage of *Taenia crassiceps*, cytosolic Trx was purified and characterized [151]. The protein is similar in its properties to the mammalian homologue. As to flukes, Trx from *Schistosoma* and *Fasciola* has been characterized [214,215,216]. It is present in the different stages of the life cycle of both parasites. Interestingly, in *S. mansoni* the protein is secreted from the eggs to the surrounding medium [216]. In *F. hepatica* Trx is expressed in both the juvenile and the adult stages. In this same species GSH is a better electron donor to Trx than its own disulfide reductase, thus making Trx similar to Grx [217]. As regards Grx, the presence of the 1-cys variant of the protein has been reported in *F. gigantica*, as well as in *S. mansoni* and *S. japonicum* [215]. The protein is outstanding in size, with 226 amino acid residues organized in two domains. The C-terminal domain shows a high identity to Trx. The redox active motif is located at the N-terminal domain. Interestingly, the presence of iron was reported in this atypical variant of Grx [218].

As regard GSH, the presence of the compound has been reported in *E. granulosus*, *T. crassiceps* and *S. mansoni*. In the cysticercus of *T. crassiceps* GSH reaches a concentration of 1 mM, with a GSH/GSSG concentration ratio of about 131 [219]. Work in this same species has revealed the importance that GSH plays under oxidative stress conditions.

In the parasite flatworms, the antioxidant enzymes include GPx, Prx and a multifunctional disulfide reductase. In *S. mansoni* a monomeric GPx has been reported, which is capable to reduce phospholipid hydroperoxides [220]. A Sec residue is critical for the catalytic function of the enzyme. In the presence of GSH as electron donor, the enzyme displays a low H_2_O_2_ reductase activity [220].

In addition to GPx, in *S. mansoni* three Cys-dependent Prx from the Prx1 subfamily have been reported: Prx1 [221], Prx2, and Prx3 [127]. The Prx1 isozyme is well represented in all the developmental stages of the parasite [127]. Although both GSH and Trx can act as electron donors, the activity with GSH is barely 10% of that with Trx [221]. As regard Prx2 and Prx3, they are able to reduce either CHP or tBuOOH with a higher efficiency as compared with Prx1. However, unlike the latter, Prx2 and Prx3 are inactivated by H_2_O_2_ [127].

In the representatives of the tapeworms sequences coding for GPx have been found in the gen bank for *T. saginata* (GenBank: OCK37178.1), *E. granulosus* (GenBank: EUB58341.1), and *E. multilocularis* (GenBank: CDS36612.2) [222,223,224]. Interestingly, in the expressed proteins a Sec residue is lacking. By contrast, in the *T. solium* genome database [210] a Sec-dependent GPx was found (TsM_000011300.1..pep). However, in no case a biochemical characterization of the enzymes has been reported. The absence of the Sec residue in some GPx from tapeworms suggests they could be mechanistically similar to the enzyme from Kinetoplastida. On the other hand, in some tapeworms species at least a 2-Cys Prx is present [225,226]. The enzyme is able to reduce either H_2_O_2_ or CHP using Trx as the reducing substrate [225]. The possibility that GSH could act as electron donor for this 2-Cys Prx has not been elucidated. It is worth to note that both *T. solium* and *T*. *crassiceps* are capable to withstand high H_2_O_2_ concentrations [226]. Interestingly, in both species the expression of Prx1 is constitutive [226].

The thiol-dependent reductase in the parasite representatives of this set of organisms are outstanding due to TGR is the only enzyme involved in the reduction of both GSSG and Trx-S_2_ [223]. The typical GR and TrxR are absent (Figure 7E), as revealed through an extensive search in the genome data-bases [210]. Hence, in this kind of parasites TGR is the only disulfide reductase to contend with ROS produced by the immune system of the host. Flatworm TGR differ from its mammalian counterpart [141] in the presence of a dithiol redox-active motif at the Grx-like domain [155,156]. The enzyme has been purified and characterized from both flukes [156,227,228,229] and cestodes [149,155,230]. TGR is located at both the cytosolic and mitochondrial compartments [152]. Although both isoforms are generated from a single gene by alternative splicing [152], they differ in their kinetic properties, perhaps influenced by the microenvironment surrounding each one [231]. Based on the results derived from immunohistochemistry studies, it has been reported the enzyme is expressed in significant levels in the male reproductive system of *F. hepatica* [232], in according with the function of TGR in mammals [157]. Similarly, in *F. gigantica* TGR reach a high level of expression in eggs, 2- and 4-week-old juveniles and adults [229].

An interesting kinetic property of TGR from parasites is the presence of a strong substrate inhibition at moderate or high GSSG concentrations [149,152,227]. The inhibition is concomitant with the observation of hysteretic-like progress curves [149]. Although the detailed molecular basis of such kinetic behavior have been not elucidated, results from the kinetics studies strongly suggests thiol-disulfide exchange reactions are involved.

Because in this kind of parasites TGR is critical for both reproductive and antioxidant purposes, the enzyme constitutes an ideal target for an anti-helminthic therapy. In the search for an effective anti-helminthic therapy, a diversity of TGR inhibitors have been tested [233,234,235]. In this sense, results with the gold-containing compound auranofin are particularly promising, because micromolar concentrations of the compound are big enough to fully inhibit in vivo TGR activity, leading to a significant decrease in parasite survival [152,236,237].

#### 5.2.2. Phylum Nematoda

This group of worms includes a significant number of parasite species of great medical and agricultural importance. However, information about its thiol-dependent antioxidant defense systems is scarce. In this sense, *Onchocerca volvulus*, *Haemonchus contortus*, *Ascaris* spp. and *Brugia malayi* have been the most studied species [25]. In *B. malayi* two Trx variants are present, which are expressed in a constitutive mode. They are distributed in the cytosolic (Trx1) and the mitochondrial (Trx2) compartments [238]. Work in this same species revealed that both, in the filarial and in the adult stages Trx1 is secreted although the physiological importance of such process remain to be elucidated. In addition to Trx, enzymes such as SOD and GPx are also secreted by the filarial stage of *B. malayi* [238]. In *H. contortus* the presence of Trx has been reported [239]. The protein can be reduced by either TrxR or GSH.

As regard the complement of antioxidant enzymes in the nematodes, the information available is also scarce. In this sense, no sequence coding for CAT from any human parasite nematode was found in the gene data bank. However, in crude extracts of *B. malayi* a low catalase enzyme activity was reported [240]. By contrast, in this same species and *Dirofilaria immitis* a secretory Cys-dependent GPx has been reported, with preferential activity toward fatty acid and phospholipid hydroperoxides [241]. In *O. volvulus*, as well as in *B. malayi*, the existence of either Prx1b or Prx1a, respectively, was reported [242,243]. Further, both in the larval and the adult stages its transcripts were found [243].

In the Nematoda, the disulfide reductase enzymes are represented by both GR and TrxR (Figure 7F). GR has been characterized from the free-living nematode *Caenorhabditis elegans* [244], as well as from the filarial *Setaria digitata* [245], *A. suum* [246], and *O. volvulus* [247]. Results derived from such studies have revealed nematode GR is very similar in both structural and catalytic properties to the mammalian enzyme. However, based on the differential response to arsenicals between filarial and human GR, the enzyme has been proposed as a potential target for filaricidal drugs [245]. As to nematodeTrxR, the enzyme has been reported from both free-living [248] and parasitic [249] species. In these organisms, TrxR is present in both the Sec-dependent and Sec-independent isoforms, which are located at cytosol and mitochondria, respectively. Interestingly, in the free-living *C. elegans* the presence of both TrxR isoforms is dispensable providing GR is present [250,251,252]. Such observation suggests GSH is critical in the maintenance of the redox state of the worm [253].

## 6. Final Comments

The central purpose of this review was to present a comprehensive summary of the state of the art of our knowledge about the thiol-based antioxidant systems in invertebrate parasites. Through the review, the diversity of redox compounds found in this kind of organisms was shown. Such diversity ranges from Cys, the building block on which the thiol-containing compounds are constructed, up to complex molecules of proteinic nature, like Trx. Figure 7 summarizes the architecture of the antioxidant redox systems both in parasite Protista and Metazoa. For comparative purposes, the composition of the corresponding system in mammals has been included (Figure 7A). In all cases, the flux of the reducing equivalents toward H_2_O_2_ arises from NADPH, passing through a reductase, a substrate-represented essentially by either GSH or Trx and then into an enzyme with peroxidase activity. However, in spite of such functional similarity, the constitution of such antioxidant systems in invertebrate parasites differs from the corresponding system in vertebrates, which represent a significant percentage of the final host of such parasites. It is worth to note that catalase, an important enzyme for H_2_O_2_ disposal in free-living organisms, is absent in all the representatives of animal parasites studied so far. In the latter, the reduction of H_2_O_2_ is mediated mainly by either the Sec- or Cys-dependent Prx. On the other hand, although the functional constitution of the antioxidant systems is similar in all the taxa analyzed, there are significant differences as to the nature of the components involved. Outstanding in this sense are the representatives of the Kinetoplastida and the Platyhelminthes, in which variants of both GSH and Trx systems are present. In the former redox compounds similar to either GSH or Trx were developed, while in Platyhelminthes a single multifunctional disulfide reductase is present.

On the other hand, from a comparison of such antioxidant systems in parasite eukaryotes, it becomes evident Trx is the only common component expressed. Although in the Kinetoplastida phylum the presence of the TrxR has not been demonstrated, its substrate Trx is present [200,201]. Even in the representatives of the primitive phylum Amoebozoa, in which the GSH system is absent, Trx is a component of its redox systems. It has been proposed the incorporation of GSH as part of the antioxidant defense systems was concomitant with the appearing of mitochondria [27,160]. The absence of functional mitochondria in Amoebozoa is consistent with such proposal. As to Sec, its presence in the antioxidant systems has been restricted to specific proteins, in spite its similar oxide-reduction properties are similar to those of Cys. Such a preference for Cys can be the result of the fact that Sec is not available as a free amino acid. In all the cases, Sec is formed from serine residues in the translation process involving a complex molecular machinery [254]. It was proposed during the evolutionary process of vertebrates a decrease in Sec utilization occurred [255]. In a similar sense, it has been proposed the transition from an aquatic to a terrestrial mode of life during the evolutionary process was accompanied by a decrease in the content of selenoproteins [256]. However, in the specific case of invertebrate parasites, no information is yet available on which similar conclusions could be reached.

On the other hand, the in silico analysis of metazoan parasites has revealed the presence of selenoproteins in both flatworm (trematodes and cestodes) [257] and roundworm [258] genomes. In *S. japonicum* (Class Trematoda) and *T. solium* (Class Cestoda) 11 and seven selenoprotein genes, were found, respectively, while in the free-living nematode *Caenorhabditis elegans* the cytosolic thioredoxin reductase is the only selenoprotein present [259]. As regard Protista, both in Kinetoplastida (such as *Leishmania* and *Trypanosoma*) [260] and Apicomplexa (*Plasmodium*) [261,262] genes coding for proteins involved in Sec-synthesis, as well as putative selenoproteins genes (six and four, respectively) have been identified. However, studies about its biochemical properties are scarce [220,227,228,229,237,251].

Finally, although not in the main concern of the present review, a final brief comment about the importance of protein turnover in the maintenance and survival of animal parasites is warranted. Such a phenomenon depends on specific cell signals and is carried out by either the apoptotic pathways, which are dependent on the activation of caspases, or ubiquitination of proteins. Caspases plays an important role in protein turnover because their activity is essential in the exposure of recognition sites on specific proteins, allowing its selective degradation [263,264]. In the degradation of proteins involving ubiquitination, the activity of the proteasome complex plays an essential role. Both systems are involved in the selective degradation of proteins, which is important in processes such as development and differentiation.

In the case of animal parasites, the selective degradation of proteins such as cyclins or transcription factors through redox signaling could lead to a modification of the specific biological cycle [265]. In this sense, in the representatives of the Kinetoplastida, the proteasome-dependent protein turnover is enhanced during the trypomastigote to amastigote transition [266], while in *T. brucei* the selective activity of the proteasome is involved in the control of cell functions in both the bloodstream and the procyclic stages of the life cycle [265]. On the other hand, in *G. lamblia* the proteasome activity is critical in the trophozoite to cyst transition [267]. Similar phenomena are known in multicellular animal parasites. Thus, in the fluke *S. mansoni* the protein turnover of specific components is essential in cercaria-schistosomula transition [268]. Detailed knowledge of the specific components of the antioxidant defense system of parasites is anticipated to lead to the successful development of design and validation of antiparasitary drugs [94].

## 7. Conclusions

The thiol-dependent antioxidant systems take advantage of the redox properties of the sulfur atom in cysteine to pass through different oxidative states. Such properties allow regulate ROS production as well as scavenge them to maintain a thiol-disulfide equilibrium in biomolecules and redox homeostasis in the cell. In obligate eukaryotic parasites the absence of catalase has been reported, so their defense against ROS is based mainly on thiol-dependent peroxidases, substrates and reductases. They are assembled in a specific antioxidant system with a particular architecture, significantly different from that observed in most of their respective hosts. Understanding the differences between the host and the parasite antioxidant systems will help us to propose new strategies to contend with parasitosis succesfully.

## Abbreviations

The following abbreviations are used in this manuscript:

2-Cys-Grx	dithiolic glutaredoxin
AhpF	alkyl hydroperoxide reductase component F
APx	ascorbate peroxidase
tBuOOH	tert butyl hydroperoxide
CAT	catalase
S_C_H	catalytic cysteine
CHP	cumene hydroperoxide
Cys	cysteine
Grx	glutaredoxin
GPx	glutathione peroxidase
GPxA	glutathione peroxidase-like tryparedoxin peroxidase
GR	glutathione reductase
GSH	glutathione (reduced form)
GSSG	glutathione disulfide (oxidized form)
H_2_O_2_	hydrogen peroxide
^.^OH	hydroxyl radical
1-Cys-Grx	monothiolic glutaredoxin
OSH	ovothiol (reduced form)
OSSO	ovithiol disulfide (oxidized form)
S_P_H	peroxidatic cysteine
ONOO^−^	peroxinitrite
Prx	peroxiredoxin
Plrx	plasmoredoxin
Plrx-(SH)_2_	plasmoredoxin (reduced form)
Plrx-(S)_2_	plasmoredoxin (oxidized form)
AOP	protein antioxidant
PDB	protein data bank
PDI	protein disulfide isomerase
ROS	reactive oxygen species
RR	ribonucleotide reductase
Rx	redoxin
S_R_H	resolving cysteine
SeOH	selenenic acid form of the Sec
SeO_2_H	seleninic acid form of the Sec
Sec	selenocysteine
-Se^−^	selenolate group
-SeH	selenol group
S_p_OH	sulfenic acid form of the peroxidatic Cys
S_p_O_2_H	sulfinic acid form of the peroxidatic Cys
S_p_O_3_H	sulfonic acid form of the peroxidatic Cys
O_2_**^.^****^−^**	superoxide anion
SOD	superoxide dismutase
-SH	thiol group
-S^−^	thiolated group
TPx	thioredoxin peroxidase
Trx	thioredoxin
Trx-S_2_	thioredoxin (ozidized form)
Trx-(SH)_2_	thioredoxin (reduced form)
L-TrxR	thioredoxin reductase (low molecular weight isoform)
H-TrxR	thioredoxin reductase (high molecular weight isoform)
TSH	trypanothione
T(S)_2_	trypanothione (oxidized form)
T(SH)_2_	trypanothione (reduced form)
TryR	trypanothione reductase
TXN	tryparedoxin
TXNPx	tryparedoxin peroxidase
